# Bacteriophage Infection of the Marine Bacterium *Shewanella glacialimarina* Induces Dynamic Changes in tRNA Modifications

**DOI:** 10.3390/microorganisms11020355

**Published:** 2023-01-31

**Authors:** Mirka Lampi, Pavlina Gregorova, M. Suleman Qasim, Niklas C. V. Ahlblad, L. Peter Sarin

**Affiliations:** 1 RNAcious Laboratory, Molecular and Integrative Biosciences Research Programme, Faculty of Biological and Environmental Sciences, University of Helsinki, FI-00014 Helsinki, Finland; 2 Doctoral Programme in Integrative Life Science, University of Helsinki, FI-00014 Helsinki, Finland; 3 Doctoral Programme in Microbiology and Biotechnology, University of Helsinki, FI-00014 Helsinki, Finland

**Keywords:** host–pathogen interaction, post-transcriptional nucleoside modification, translation, transfer RNA, Shewanella phage 1/4, *Shewanella glacialimarina*

## Abstract

Viruses are obligate intracellular parasites that, throughout evolution, have adapted numerous strategies to control the translation machinery, including the modulation of post-transcriptional modifications (PTMs) on transfer RNA (tRNA). PTMs are critical translation regulators used to further host immune responses as well as the expression of viral proteins. Yet, we lack critical insight into the temporal dynamics of infection-induced changes to the tRNA modification landscape (i.e., ‘modificome’). In this study, we provide the first comprehensive quantitative characterization of the tRNA modificome in the marine bacterium *Shewanella glacialimarina* during Shewanella phage 1/4 infection. Specifically, we show that PTMs can be grouped into distinct categories based on modification level changes at various infection stages. Furthermore, we observe a preference for the UAC codon in viral transcripts expressed at the late stage of infection, which coincides with an increase in queuosine modification. Queuosine appears exclusively on tRNAs with GUN anticodons, suggesting a correlation between phage codon usage and PTM modification. Importantly, this work provides the basis for further studies into RNA-based regulatory mechanisms employed by bacteriophages to control the prokaryotic translation machinery.

## 1. Introduction

Virus infection constitutes a complex and delicately orchestrated interplay between cellular and viral components [1]. Since all viruses are obligate parasites and fully dependent on cellular translation, obtaining translational control is key to their replication. Viruses utilize a variety of mechanisms to achieve this, including both virus-encoded factors and skillful exploitation of cellular protein and RNA components to commandeer the translation machinery [1,2].

Within the translation machinery, transfer RNA (tRNA) molecules are crucial amino acid-carrying adaptor molecules that decode the codon triplets to form polypeptide chains. These tRNA molecules are highly decorated with post-transcriptional nucleoside modifications (PTMs) that, depending on their location and chemical nature, may have various effects on the molecule and its function in translation [3]. Core modifications often provide structural stability and aid the correct folding of the tRNA clover-leaf structure [4]. They are also essential markers for aminoacyl tRNA transferases, enabling them to distinguish and charge tRNAs with their correct cognate amino acids [5]. Modifications at the tRNA anticodon stem loop (ASL), which recognizes and base pairs with the messenger RNA (mRNA) codons, are critical determinants of the rate and fidelity of translation. The ASL modification hotspots include the first base at the anticodon triplet, the so-called ‘wobble’ position 34, and the 3′ adjacent position 37. Position 34 modifications enable tRNA coding capacity expansion via non-Watson–Crick base pairing, but they may also restrict base pairing to explicit target codons, whereas position 37 modifications further reading frame maintenance [3,6].

Viruses have evolved to utilize various RNA components, including tRNA modifications, to further their replication. For example, the nucleocapsid of the human immunodeficiency virus (HIV) selectively binds to 5-methoxycarbonylmethyl-2-thiouridine (mcm^5^s^2^U_34_) and 2-methylthio-*N*(6)-threonylcarbamoyladenosine (ms^2^t^6^A_37_) modified tRNA$\begin{array}{c} \mathrm{UUU}\\ \mathrm{Lys}3 \end{array}$, thereby furthering viral packaging and priming of the reverse transcriptase enzyme [7]. This modification-based recognition mechanism has been suggested as a potential target for novel antivirals to suppress HIV replication [8], thus highlighting the importance of PTMs in the viral life cycle.

Furthermore, many host stress-response genes and regulons as well as viral genomes are encoded using codon triplets that differ from the anticodon availability in the cellular tRNA pool [9,10]. To counteract this imbalance, critical bacteriophage proteins that are expressed in large quantities during infection, such as the major capsid protein (MCP), tend to be more adapted to host codon usage [11]. To further ensure efficient translation, dynamic changes to the PTM levels are required to expand the decoding capacity of the tRNAs. Such translation-level regulation of stress responses has been well-established for many organisms [12,13], and the same mechanisms may be exploited by viruses as well. A recent study by Jungfleisch et al. showed that Chikungunya virus infection increases the abundance of 5-methoxycarbonylmethyluridine (mcm^5^U_34_) modification, thus favoring the translation of A-ending codons preferred by the Chikungunya genome and enhancing virus replication [14]. Moreover, host cells may also utilize changes in PTM levels to perturb virus infection. For instance, loss of 2-thiolation causes ribosomal frameshifting in *Escherichia coli*, which skews the ratio at which the Escherichia phage λ proteins gpG and gpGT are expressed and thus hinders efficient virus replication [15].

Despite this, it is not well understood how viruses, and in particular bacteriophages, utilize tRNA modifications at various stages of the infection cycle. In this study, we utilize quantitative liquid chromatography–mass spectrometry to characterize how Shewanella phage 1/4 infection alters the dynamic nature of the tRNA modification landscape (i.e., ‘modificome’) throughout the infection cycle in the cold-active marine bacterium *Shewanella glacialimarina*. This host–virus model is of particular interest for tRNA modification studies as the Shewanella phage 1/4 genome contains two virus-encoded tRNA (vtRNA) genes, vtRNA$\begin{array}{c} \mathrm{UCU}\\ \mathrm{Arg} \end{array}$ and vtRNA$\begin{array}{c} \mathrm{CCU}\\ \mathrm{Gly} \end{array}$, and one putative vtRNA gene [16]—which may all partake in translation. We show that Shewanella phage 1/4 has a short intracellular life cycle during which significant rearrangements in cell morphology occur. Furthermore, we observe a likely correlation between MCP codon usage bias and increased queuosine levels at the late stage of infection, suggesting a potential regulatory function for this tRNA modification. This modificome analysis provides the basis for further exploratory work on phage-induced PTM-based translational regulation in prokaryotes.

## 2. Materials and Methods

### 2.1. Bacterial Strains and Their Growth

Cultivation of the host bacterium, *Shewanella glacialimarina* TZS-4_T_ (CP041216.1), was conducted as previously described by Qasim et al. [17]. Strain TZS-4_T_ was grown on 25% rich Marine Broth (rMB) agar plates over two nights at 15 °C until colonies appeared. For liquid starter cultures, a single colony was inoculated into 30 mL of 25% rMB and grown at 15 °C with 200 rpm aeration over two nights. Starter cultures were used to inoculate 25% rMB to reach an optical density of 0.2 at wavelength 600 nm (OD_600_). The *S. baltica* (LMG2250) and *S. frigidimarina* (ACAM 591) reference strains used for the host range study were obtained from the Leibniz Institute DSMZ collection of microorganisms (cat. no. 85DSM-9439 and DSM-12253, respectively). For *S. glacialimarina*, colonies and starter cultures were grown at 22 °C, although for a shorter duration (over one night).

### 2.2. Production and Purification of the Bacteriophage

Shewanella phage isolate 1/4 (hereon referred to as phage 1/4) was initially isolated from Baltic Sea ice close to Tvärminne zoological station, Hanko, Finland [18]. In brief, the agar stock of the virus was prepared as follows: Part of the original bacteriophage stock (stored at −80 °C) was resuspended in 25% rMB. A 100 µL aliquot of the 10^−5^ dilution, which yields semiconfluent plates, was plated with 100 µL of the host starter culture and 3 mL of 25% rMB soft agar (contains 7.5 g agar/L). Plates were incubated at 15 °C for two nights, after which the top layer was collected and mixed with 2.5 mL/plate of 25% rMB media with 200 rpm shaking. Viruses were separated from agar and cell debris using centrifugation (7000× *g*, 15 min, 4 °C). The resulting agar stock was filtered through 0.45 µm and 0.22 µm filters and stored at 4 °C.

Phage 1/4 was purified as described by Luhtanen et al. [18]. *S. glacialimarina* liquid culture was grown to the early logarithmic growth phase (OD_600_ ≈ 0.6) and cells were infected using a multiplicity of infection (MOI) value of 10. After cell lysis, the debris was pelleted using centrifugation (8330× *g*, 15 min, 15 °C). The phages were precipitated from the lysate using 10% (*w*/*v*) polyethylene glycol (PEG) 6000 and 0.5 M NaCl and collected using centrifugation (8330× *g*, 40 min, 4 °C). The precipitated particles were resuspended in SM buffer [50 mM Tris pH 7.5, 100 mM NaCl, 8 mM MgSO_4_, 0.01% gelatin (*w*/*v*)] at ~1/100 of the initial volume. The virus preparate was further purified on a 10–30% (*w*/*v*) sucrose gradient (prepared in SM buffer) using rate-zonal ultracentrifugation (Sorvall rotor AH629, 103,400× *g*, 25 min, 10 °C). The light-scattering virus zone was collected and concentrated using differential centrifugation (113,580× *g*, 3 h, 10 °C). This final virus preparate is referred to as the 1× purified virus (1× virus).

### 2.3. Host Range Assessment

The host range of the Shewanella phage 1/4 was investigated using *S. baltica* and *S. frigidimarina* as potential hosts on both soft agar plates and in liquid culture. A 100 µL aliquot of the host starter culture mixed with 3 mL of 25% rMB soft agar was plated on a 25% rMB plate and grown until the bacterial lawn was formed. A droplet (~40 µL) of an agar stock, PEG-precipitated virus preparates, or 10^−2^ diluted 1× virus was spotted on the lawn (i.e., spot-on-lawn test), the plates were incubated at 22 °C for two nights, and changes in the lawn were monitored. Host range assessment in liquid cultures were performed by infecting early logarithmic growth phase cultures (OD_600_ ≈ 0.6) using MOI 10 and monitoring the growth as changes in OD_600_ at regular intervals.

### 2.4. Adsorption Time and Receptor Saturation

To establish the adsorption time, aliquots of early-logarithmic growth phase *S. glacialimarina* cells (á 0.650 mL ≈ 3 × 10^8^ cfu) were infected using 1.5 × 10^3^ virions and mixed well. Next, cells were removed using centrifugation (11,300× *g*, 5 min, 4 °C) from an infected aliquot at 0, 5, 10, 15, or 20 min post-infection (p.i.). To assess the amount of residual (unadsorbed) phages in the growth media, 50 µL of the supernatant from each time point was plated with the host bacterium starter culture, as described in Section 2.2. Plates were incubated at 15 °C for two nights, after which the number of plaques obtained was compared to that of the control (0.650 mL of 25% rMB and the same amount of the bacteriophages). This mimics the situation where no phages adsorb whilst accounting for the possible centrifugation-caused effects on adsorption efficiency. Finally, the adsorption rate constant (*k*) was calculated using the formula *k* = (2.3/Bt) × log(p0/p), where p0 and p represent free virus concentrations (pfu/mL) at the time point zero and after time t (here 10 min), respectively, while B represents the concentration of the cells (cfu/mL) [19].

Early logarithmic growth phase cells were infected using MOI 1, 5, 10, 30, and 50. As a control that simulates a situation where no phages are adsorbed, the equivalent amount of virus was added to 25% rMB. After a 10 min adsorption period, the cells were harvested (11,300 g, 5 min, 4 °C), and the number of free viruses in the supernatant was assessed using plaque assay and comparing the infected samples to the controls.

### 2.5. Infection Cycle

For an initial characterization of the infection cycle in 25% rMB and to determine the optimal MOI value, *S. glacialimarina* liquid cultures were infected at the early logarithmic growth phase (OD_600_ ≈ 0.6) using a range of MOI values (0.2–10). The growth was monitored by measuring the OD_600_ of the culture.

Early logarithmic growth phase cells in liquid culture were subsequently infected using MOI 10. Phages were let to adsorb for 10 min, after which the cells were washed twice with 25% rMB using centrifugation (3220× *g*, 5 min, 4 °C) and resuspended back to their original volume in 25% rMB. Infected cultures were grown at 15 °C with 200 rpm aeration. Samples of 1 mL were collected at 15, 30, 60, 120, and 180 min p.i., as well as prior to washing. Cells were harvested using centrifugation (6700× *g*, 5 min, 4 °C). The supernatants were collected and titrated to estimate the number of free viruses in the culture.

To visualize infection-induced changes in the protein content of the growth media and infected cells, proteins from the abovementioned cell pellets and supernatants were analyzed on 10% Tris-Glycine-SDS-PAGE. Proteins from the supernatant samples were precipitated by adding 10% (*v*/*v*) cold trichloroacetic acid (TCA) and incubating on ice for 30 min. The precipitate was collected using centrifugation (11,300× *g*, 30 min, 4 °C), and the protein pellets were dissolved in 125 µL of 1× Laemmli loading buffer (62.5 mM Tris pH 6.8; 10% (*v*/*v*) glycerol; 2% (*w*/*v*) SDS; 2% (*v*/*v*) β-mercaptoethanol; 0.1% (*w*/*v*) bromophenol blue). Cell pellets were resuspended in 200 µL Tris-HCl buffer (pH 7.2) and mixed with 66.6 µL of 4× Laemmli loading buffer. All samples were heat-denatured at 95 °C for 2 min and loaded onto the gel in 20 µL volumes. The Unstained Page Ruler ladder (Thermo Scientific, 26630, Waltham, MA, USA) and 5 µL of 1× virus were applied as size markers. Gels were stained with Coomassie blue and visualized using a ChemiDoc MP Imaging System (BioRad, Hercules, CA, USA).

### 2.6. Electron Microscopy

The cell morphology and surface structures were visualized using scanning electron microscopy (SEM). Briefly, cells were grown to the early logarithmic phase and 10 mL of the culture was infected using MOI 10 or mock infected with an equal volume of 25% rMB. Cells were fixed 2 min p.i. with 2.5% (*v*/*v*) glutaraldehyde (Sigma) at 4 °C for 20 h, harvested (3200 g, 5 min, 4 °C), washed with 10 mL of PBS, and finally resuspended in 5 mL of PBS. The final samples for microscopy were prepared as previously described [17] and analyzed using a FEI Quanta 250 Field Emission Gun (FEG) Scanning Electron Microscope.

For intracellular views, thin-section cuts of MOI 10 infected and mock-infected *S. glacialimarina* cells were prepared. After a 5 min adsorption time, unattached phages were removed using centrifugation (3220× *g*, 10 min, 4 °C) and the cells were resuspended to the original volume in 25% rMB. Samples were collected at 0, 15, 30, 60, and 120 min p.i. Cells were fixed by adding 2.5% (*v*/*v*) glutaraldehyde followed by an incubation at RT for 40 min and then overnight at 4 °C, respectively. To remove the glutaraldehyde, cells were collected (3220× *g*, 10 min, 4 °C) and resuspended in the original volume of 25% rMB and, after another centrifugation as above, resuspended in 1/3 of the original volume in PBS. Thin sectioning was conducted at the Electron Microscopy Unit (Institute of Biotechnology, University of Helsinki) by post-staining with osmium tetroxide, ethanol dehydration (70, 96, and 100%), incubation with transitional solvent acetone, and embedding in Epon. Thin sections were cut using a Leica EM Ultracut UC6i ultramicrotome.

Negative-stained samples on Cu mesh grids were prepared using the infected cells and the 1× virus. Early logarithmic growth phase cells were infected using MOI 50. After 1 min of adsorption, 2 µL of the culture was incubated on a carbon-coated Cu mesh grid for 1 min and negative stained with 2% uranyl acetate for 15 s. To visualize the phage, 1× virus was diluted 1/1000 in SM-buffer, of which 2 µL was incubated on the grid and treated similarly as described above.

Transmission electron microscopy (TEM) was performed using a JEOL JEM-1400 microscope (Jeol Ltd., Tokyo, Japan) operating at 80 kV and equipped with Gatan Orius SC 1000B bottom-mounted CCD camera (Gatan Inc., Pleasanton, CA, USA).

### 2.7. RNA Isolation

For RNA isolation, 50 mL cultures were infected (MOI 10), and cells were harvested at 0, 15, 30, 60, 120, and 180 min p.i. The harvested cell pellet was resuspended in 10 mL of Trizol. Glass beads and 1 mL of 1-bromo-3-chloropropane (BCP) were added, and then the mixture was vortexed thoroughly and centrifuged (10,000× *g*, 15 min, 22 °C). The RNA was further re-isolated from the aqueous phase with the addition of 2 mL of acidic phenol, 400 µL of BCP, and using centrifugation (10,000× *g*, 15 min, 22 °C), after which the RNA was precipitated from the aqueous phase with 99.6% ethanol. The RNA pellets were air-dried and dissolved in double-distilled water (ddH_2_O).

To extract tRNA, the volume of the total RNA samples was adjusted to 10 mL with equilibration buffer (10 mM Tris-HCl, pH 6.3, 15% ethanol, 200 mM KCl) and applied onto a pre-equilibrated (with equilibration buffer containing 0.15% Triton X-100) Nucleobond AX-100 column (Macherey-Nagel). The column was washed twice with wash buffer (10 mM Tris-HCl pH 6.3, 15% ethanol, 300 mM KCl), and tRNA was eluted with warm (55 °C) elution buffer (10 mM Tris-HCl pH 6.3, 15% ethanol, 750–800 mM KCl) into 2.5 vol. of 99.6% ethanol. The eluted tRNA was precipitated over night at −20 °C and pelleted using centrifugation (10,000× *g*, 4 °C for 30 min). Residual salt was removed with consecutive 80% ethanol washes. The final tRNA pellet was air-dried and resuspended in ddH_2_O. The quality and purity of the isolated tRNA was assessed with denaturing electrophoresis on 10% 8 M urea-polyacrylamide gels.

### 2.8. Codon Usage Analysis

*S. glacialimarina* and phage 1/4 genomes were downloaded from GenBank with accession codes NZ_CP041216.1 and NC_025436.1, respectively. The phage 1/4 genome was re-annotated using Prokka v. 1.14.6 [20] with the default settings for annotating viruses to exclude noncoding regions from the following analysis. Next, codon usage analysis was conducted for all coding sequences with the codon usage tool from The Sequence Manipulation Suite [21]. Furthermore, the codon usage was also analyzed for the following genes: a set of viral early genes including DNA polI (GeneID: 22110575), DNA primase (GeneID: 22110576) and RNAseHI (GeneID: 22110577), a set of viral late genes containing the major capsid protein (MCP) (GeneID: 22110506), tail sheet protein (GeneID: 22110539), tape measure protein (GeneID: 22110542) and tail assembly chaperone (GeneID: 22110541), and the MCP gene separately.

### 2.9. Ultraperformance Liquid Chromatography–Mass Spectrometry (UPLC-MS) Analysis of tRNA Modifications

Dephosphorylated monoribonucleosides were prepared as previously described [22]. Ribonucleoside standards and samples (500 ng) were separated and detected identically to the previously described C18-UPLC method [23]. MS data were analyzed in MZmine2 (version 2.52) and exported in csv format [24]. Peak identification was performed with the custom database identification module of the program using a custom lookup list of modified ribonucleosides compiled from Modomics [25] data and retention times acquired from analyzing the standards. The absolute intensities were internally normalized to the signal obtained for adenosine. The relative changes in ribonucleoside modifications are presented as ratios of normalized intensities between infected samples and non-infected controls.

### 2.10. Statistical Analyses

Error bars in plots represent standard deviations of three independent biological replicates and *p*-values were calculated using a one-sample Student’s *t*-test. The data visualization and calculations of the heatmap were conducted in R (version 3.6.3) using the pheatmap package (version 1.0.12).

## 3. Results

### 3.1. High MOI Is Required for Complete Infection of the Liquid S. glacialimarina Culture

A previous study reported that phage 1/4 has a lytic infection cycle, as well as a putative abortive infection mechanism that is triggered when the MOI value exceeds 0.2 [16]. However, this low MOI value implies that the cultures were not uniformly infected, which might have affected the progression of the infection cycle. Moreover, this study was carried out with undefined Zobell growth media based on aged Baltic Sea water [18,26]. We have previously shown that *S. glacialimarina* grows well in the defined 25% rMB [17], so we set out to recharacterize the infection characteristics in this new growth medium. First, we conducted infection cycle studies using an MOI ranging from 0.2 to 10 (Supplementary Figure S1). Each virus amount caused significant growth retardation of the host cells, but the duration of the infection cycle varied (Supplementary Figure S1). An initial reduction in turbidity of the bacterial culture was observed at 30 min p.i. for MOI values 4–10. This might be accounted for by cells entering apoptosis upon infection-induced stress, and viral infection is also likely to cause transcriptional and translational reprogramming, manifesting as a slowdown of growth. A clearly noticeable decrease in turbidity, i.e., cell lysis, was observed at ~150 min p.i. when an MOI of ≤ 2 was used but increasing the MOI to 4–10 resulted in lysis occurring more uniformly at ~120 min p.i. (Supplementary Figure S1).

Amongst the bacterial strains isolated in the original study, phage 1/4 was found to solely infect *S. glacialimarina* [18]. To investigate whether the host range might also include other closely related *Shewanella* strains, we attempted to infect both *S. baltica* and *S. frigidimarina* with phage 1/4. However, we did not observe any visible changes in the spot-on-lawn tests for these potential hosts (Supplementary Figure S2A,B). Likewise, no growth retardation or cell lysis was observed in exponential growth phase liquid cultures infected with an MOI of 10 (Supplementary Figure S2C), demonstrating that phage 1/4 infection could not be established in *S. baltica* and *S. frigidimarina*.

**Figure 1:**
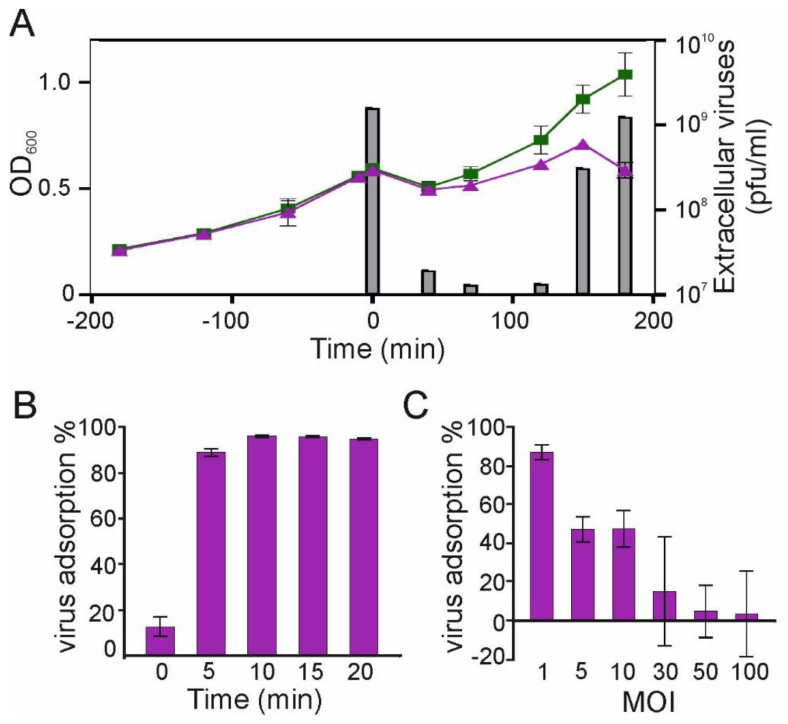
Characterization of phage 1/4 infection. (**A**) Growth curves of MOI 10-infected and mock-infected *S. glacialimarina*. The columns depict the number of extracellular viruses (pfu/mL) at different time points of the infection. (**B**) Shewanella phage 1/4 adsorption dynamics following the addition of a fixed number (1.5 × 10^3^) of virions at t_0 min_. (**C**) Host receptor saturation assay showing phage 1/4 adsorption at t_10 min_ in response to increasing MOI values. Error bars show the standard deviation (*n* = 3).

Next, we determined the length of the intracellular infection cycle of phage 1/4 in *S. glacialimarina* by monitoring the number of free viruses in the growth media (Figure 1A), as well as the intra- and extracellular viral protein contents (Supplementary Figure S3). Samples collected from the growth media revealed extracellular viral proteins appearing at 120 min p.i., indicating that the release of virions from the cells coincides with cell lysis (Supplementary Figure S3). A closer inspection of the protein pattern obtained from the infected cells showed that intracellular viral proteins could not be detected at 40 min p.i., but they were present at 100 min p.i. (Supplementary Figure S3). To corroborate these results, we performed a phage titration assay of the growth media throughout the infection cycle. This confirmed that the number of extracellular virions decreased when all unbound phages were removed with washing after the adsorption step, and extracellular virions only reappeared in the media once the infection cycle was completed and the cells lysed, thus releasing the virus progeny (Figure 1A).

Upon successful attachment and entry of the virus into the host cell, the intracellular replication cycle commences. To ascertain the duration of the attachment and entry phase, we performed an adsorption assay, which revealed that the maximal adsorption was reached at 10 min p.i. (Figure 1B). The adsorption rate was determined to be *k* = 2.2 × 10^−9^ phage/cell/mL/min (Figure 1B), which is comparable to other bacteriophages isolated from cold marine environments [16,27]. To further evaluate the adsorption characteristics, we also performed a receptor saturation assay. With an MOI of 1, the adsorption neared completion as 87% of the virions were adsorbed, whereas when infecting with MOI 5 or 10, approximately half of the virions adsorbed, indicating that the available attachment sites were saturated. Indeed, when applying an MOI of 30, most of the virions remained free in the media (Figure 1C). Hence, a sufficiently high MOI value is required to ensure synchronized infection and thus, a uniform progression of the infection cycle. Importantly, we did not observe abortive infection for any MOI tested in this study when using the defined 25% rMB growth media. Based on these results, we decided to proceed with MOI 10 and an adsorption time of 10 min to ensure complete and uniform infection of cultures for subsequent tRNA modification analysis.

### 3.2. Phage 1/4 Infection Causes Comprehensive Changes to Intra- and Extracellular Structures

**Figure 2:**
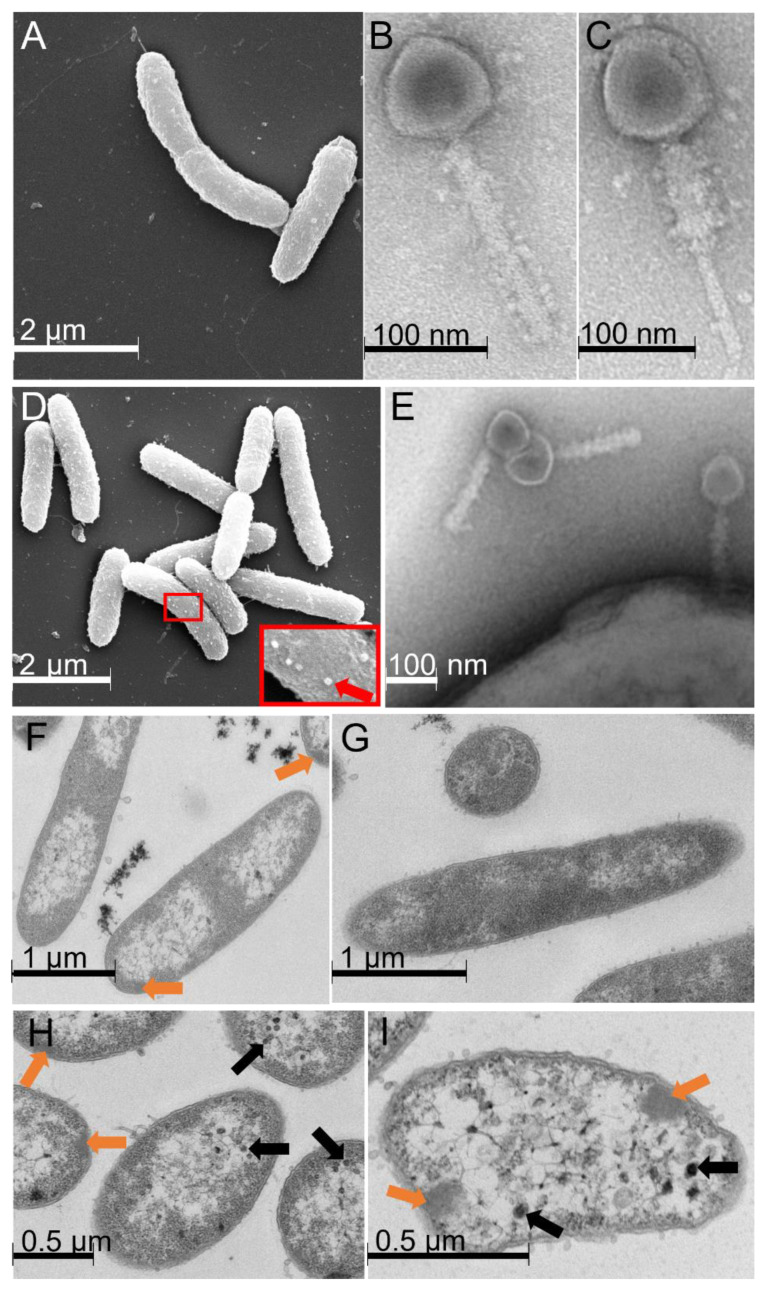
Electron microscopy micrographs of phage 1/4 and its host, *Shewanella glacialimarina***.** SEM micrograph of (**A**) *S. glacialimarina* and TEM micrographs of phage 1/4 with (**B**) non-contracted and (**C**) contracted tail conformations. SEM micrograph of (**D**) *S. glacialimarina* infected with phage 1/4. The close-up shows phage particles attached to the cell surface (indicated with a red arrow) and a TEM micrograph of (**E**) phage 1/4 attached to the cell surface. Thin-layer sections of infected (**F**) and mock-infected (**G**) *S. glacialimarina* cells at 30 min p.i., and infected cells at (**H**) 60 min p.i. and (**I**) 120 min p.i. Orange arrows indicate the denser patches forming in infected cells and black arrows show intracellular capsids of viruses.

The host cells displayed a rod-shaped morphology, as previously described by Qasim et al. [17]. In addition, a fraction of the cells expressed a single polar flagellum (Figure 2A). *Shewanella glacialimarina* phage 1/4 belongs to the class Caudoviricetes and features a typical myovirus morphotype [18]. The head–tail length of the phage is ~215 nm and the diameter of the head is ~80 nm (Figure 2B). We observed the phage 1/4 tail both in its extended and in its contracted conformation, which is a common feature of myoviruses. In the contracted form, the tail sheath length was reduced to ~75 nm (Figure 2B,C). Using SEM, we could observe phages on the surface of infected cells (Figure 2D). Intriguingly, phage 1/4 infection forms clustered aggregates on the cell’s surface (Figure 2D), possibly due to disruption of the extracellular surface matrix (ECM). Such changes might be associated with phage-induced hydrolysis of the ECM, which is thought to mask surface receptors used for virus entry and thereby prevent infection. Furthermore, this could also constitute a bacterial mechanism to avoid infection [28]. The phages were attached directly to the cell surface but not to the flagellum (Figure 2E), which is a known attachment route for many bacteriophages [29].

An intracellular visualization of the infected cells revealed massive rearrangements and disruption of the cellular contents, which began at 15 min p.i. and became more prominent as the infection proceeded. We observe that centrally located parts of the infected cells become void from 15 min p.i. onwards, leaving only the regions close to the cell wall filled with cellular material (Figure 2F,H,I, Supplementary Figure S4), whereas mock-infected cells do not display any changes (Figure 2G, Supplementary Figure S4). At 30 min p.i., denser patches right beneath the cell surface start to emerge in the infected cells (Figure 2F), and from 60 min p.i. onwards, bacteriophage capsids are seen alongside these patches (Figure 2H,I). At 120 min p.i., the fraction of infected cells that remain intact largely resemble empty shells that accommodate virus capsids and the infection-induced dense patches (Figure 2I). However, cells fixed immediately after infection were similar in appearance to mock-infected cells, for which no changes were observed during the 120 min timeframe monitored here (Supplementary Figure S4).

### 3.3. Codon Usage Analysis Reveals Unexpected Mismatches between Phage 1/4 and Its Host

Phages frequently have a somewhat divergent codon usage when compared to the host genome. Moreover, viral gene expression is strictly regulated according to the infection phase, as genes expressed during early infection have different codon usage patterns than genes expressed in the later stages of infection [30]. As codon usage differences may have a regulatory role in the *S. glacialimarina* phage 1/4 replication cycle, we compared the codon preferences of the host and the virus. We analyzed all coding sequences (CDS) as well as gene sets expressed at the early or late stages of the infection cycle [30]. In addition, the gene encoding for the major capsid protein (MCP) was separately analyzed, as it is expressed in high abundance and often features a codon usage similar to the host genome [31].

The analysis of all coding sequences of the host and the virus uncovered slight differences in codon preferences for two amino acids: proline (CCT) and serine (AGT) (Table 1). A more profound difference was observed for cysteine (TGT), where the host genome utilizes both available codons with only a slight preference for TGC, whereas the viral genome is strongly biased towards the TGT codon (Table 1, Supplementary Table S1). In addition, the codon usage of the MCP gene, which is expressed at the late stage of infection, revealed slight differences for phenylalanine (TTC) but clear differences for isoleucine (ATC), leucine (CTA), and tyrosine (TAC) (Table 1). Remarkably, arginine (CGT) was almost exclusively utilized in the MCP gene, but it was not the preferred codon for either the host or the phage.

The set of three viral late genes analyzed here showed a similar codon bias as that of the MCP, although the difference to the host genome was less evident. In addition, a slight bias towards isoleucine (ATA) and lysine (AGG) could be seen. However, for the set of genes expressed early in infection [30] (Table 1), only two changes were observed—a clear bias for cysteine (TGT) and a slight difference in the usage of serine (AGT).

Notably, the phage 1/4 genome contains two tRNA genes encoding for isodecoder tRNA$\begin{array}{c} \mathrm{UCU}\\ \mathrm{Arg} \end{array}$ and isoacceptor tRNA$\begin{array}{c} \mathrm{CCU}\\ \mathrm{Gly} \end{array}$ [16]. Of these, tRNA$\begin{array}{c} \mathrm{UCU}\\ \mathrm{Arg} \end{array}$ match the highest preference in both the host and the phage genome, although it is barely utilized in the MCP transcript. This might suggest that tRNA$\begin{array}{c} \mathrm{UCU}\\ \mathrm{Arg} \end{array}$ is essential during normal growth and at early stages of infection, whereas its importance diminishes as infection proceeds. This hypothesis is supported by the fact that the host only has one copy of the tRNA$\begin{array}{c} \mathrm{UCU}\\ \mathrm{Arg} \end{array}$ gene. Thus, the virus-encoded second gene copy would ensure sufficient availability of tRNA$\begin{array}{c} \mathrm{UCU}\\ \mathrm{Arg} \end{array}$ at the early infection stage. On the other hand, tRNA$\begin{array}{c} \mathrm{CCU}\\ \mathrm{Gly} \end{array}$ is not the preferred codon in either genome, neither in the MCP gene nor in the early or late gene sets (Supplementary Table S1). Hence, as tRNA$\begin{array}{c} \mathrm{CCU}\\ \mathrm{Gly} \end{array}$ is only sparingly utilized, it is possible that the virus-encoded copy has a role in controlling co-translational folding and translation rate, or that it partakes in non-canonical regulatory functions in the cell [32].

### 3.4. Phage 1/4 Infection Alters Host tRNA Modification Dynamics to Favor Virus Replication

**Figure 3:**
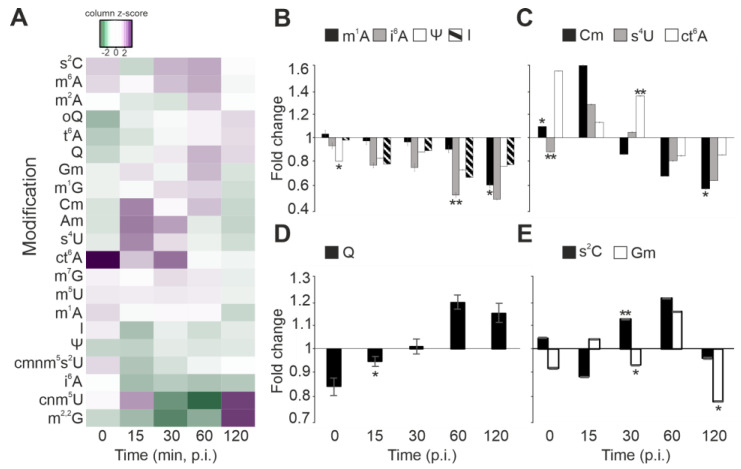
tRNA modification dynamics during infection in *S. glacialimarina***.** (**A**) Heatmap presentation of the 18 modifications detected in *S. glacialimarina* throughout the infection cycle. The tRNA modification levels in infected cells are depicted as relative fold changes to the mock-infected controls. (**B**–**E**) Selected modifications grouped according to change in abundance relative to the infection stage; (**B**) decreasing trend, (**C**) initial upregulation followed by a decrease, (**D**) increasing trend, and (**E**) fluctuating modification levels. Error bars indicate standard deviation (*n* = 3). * = *p* < 0.05, ** = *p* < 0.07.

As the codon usage analysis revealed an interesting dichotomy for specific viral genes, we next decided to investigate how post-transcriptional tRNA modifications, which are known modulators of translation during stress, are altered during the infection cycle. To this end, we performed a quantitative C18-UPLC-MS [23] analysis and detected 18 modifications that were consistently present in all samples. Furthermore, none of the modifications disappeared, nor did any new modification appear in the infected or mock-infected cells throughout the infection cycle (Figure 3A).

The host tRNA modifications can be grouped into four categories based on their response to infection. First, a majority of the detected tRNA modifications decreased in prevalence as infection progressed. This can be clearly observed for, e.g., 1-methyladenosine (m^1^A) and pseudouridine (Ψ), which are often considered important for maintaining the stability and correct fold of the tRNA molecule (Figure 3B) [33,34]. However, also the ASL modifications inosine (I) and isopentenyl adenosine (i^6^A) became less abundant (Figure 3B). I_34_ is associated with translational control via modulation of the codon–anticodon interaction, whereas i^6^A_37_ stabilizes codon recognition by stacking interactions of the first Watson–Crick base pair [35].

Second, the levels of 2′-O-methylcytidine (Cm), 4-thiouridine (s^4^U), and cyclic *N*6-threonylcarbamoyladenosine (ct^6^A) were upregulated in the first half of the infection cycle but decreased as the infection progressed (Figure 3C). Cm is an abundant ribose modification, which can be located either in the tRNA D-loop at position 18 or at ASL positions 32 and 34 [36]. Importantly, Cm_34_ stabilizes the duplex formed with the complementary RNA and increases the efficiency to read G-ending codons [37]. In addition, Cm has been linked to oxidative stress responses, which trigger similar host response pathways as those induced by virus infections [36,38]. Furthermore, 4-thiouridine (s^4^U) has been linked to tRNA quality control, as s^4^U hypomodification leads to rapid tRNA degradation by the RNA degradosome in *Vibrio cholerae* [39]. *N*6-threonylcarbamoyladenosine (t^6^A) and its derivates are located at position 37 of tRNA isoacceptors coding for ANN codons. As t^6^A enhances anticodon–codon base-pairing, a high abundance of ct^6^A at the early infection stage might indicate a possible role as a modulator in the expression of non-structural viral proteins [40].

Third, queuosine (Q) displayed an opposite profile, being initially downregulated at the early stages of infection but increased in prevalence as infection progressed (Figure 3D). This trend is particularly interesting, since Q is located at the ASL ‘wobble’ position 34 of GUN coding tRNAs, and it has previously been associated with enhancing the translation of U-ending codons [41]. Notably, our codon usage analysis uncovered that tRNA$\begin{array}{c} \mathrm{GUA}\\ \mathrm{Tyr} \end{array}$ is the preferred codon utilized in Shewanella phage 1/4 late genes, particularly of the MCP gene, which needs to be expressed in high quantities. Intriguingly, tRNA$\begin{array}{c} \mathrm{GUA}\\ \mathrm{Tyr} \end{array}$ is Q_34_ modified, and a strong preference towards this codon correlates with our observation that Q levels increase towards the late stage of phage 1/4 infection (Figure 3D).

Fourth, the final group of modifications including 2-thiocytidine (s^2^C) and 2′-O-methylguanosine (Gm) showed fluctuating modification levels between the sampled time points (Figure 3E). Indeed, s^2^C was upregulated during infection, except for the 15 min and 120 min p.i. time points, whereas Gm showed reduced levels right after infection, at 30 min p.i., as well as at 120 min p.i. The s^2^C_32_ modification in tRNA$\begin{array}{c} \mathrm{ICG}\\ \mathrm{Arg} \end{array}$ has been reported to decrease the translation efficiency of this codon, thus optimizing translation speed in relation to translation fidelity [42].

## 4. Discussion

The cold-active marine bacterium *Shewanella glacialimarina* is well-adapted and highly tolerant to the fluctuating temperature and salinity conditions of the Baltic Sea [17]. This bacterium is also the only known host for Shewanella phage 1/4, a lytic myovirus that carries two tRNA-encoding genes of undetermined function [16]. Since viruses utilize various strategies including RNA components to control the host translation machinery, we set out to investigate how phage 1/4 infection alters *Shewanella glacialimarina* tRNA modification levels at various stages of the infection cycle. In this study, we have shown that the complete phage 1/4 infection cycle, which includes virus adsorption, entry, replication, virion assembly, and release of new progeny virions, takes ~120 min (Figure 1). Interestingly, we observed a massive rearrangement of cellular contents already at 15 min p.i. (Figure 2, Supplementary Figure S4), suggesting an ongoing intracellular replication stage. This implies that the adsorption time was short (Figure 1B) and phage attachment and entry to the cells proceeded immediately following the addition of the virus. The onset of the late infection stage is often demarcated by the production of viral structural proteins. We noted assembled phage 1/4 capsids in infected cells at 60 min p.i. (Figure 2H), whereas viral proteins could not be observed within the detection limit of Coomassie-stained polyacrylamide gel analysis at 40 min p.i. (Supplementary Figure S3). Hence, this implies that early gene expression events, i.e., those preceding the 60 min p.i. time point, establish phage 1/4 infection and genome replication, whereas subsequent progeny virus production and maturation take place until the cells lyse at ~120 min p.i.

Viral genes expressed at the early stage of the replication cycle often feature a codon usage that closely matches that of the host genome. This ensures a fast and efficient translation of enzymes required for virus genome replication, such as the DNA polymerase, helicase, and DNA primase [30,43]. Our codon usage analysis revealed that early genes in phage 1/4 are highly similar and feature little codon bias compared to the host genome (Table 1). On the other hand, the set of late genes showed a significantly higher degree of codon bias and, the MCP gene in particular, preferentially utilizes the tyrosine (TAC) codon, whereas the host genome strongly favors tyrosine (TAT) (Table 1, Supplementary Table S1). This discrepancy in codon usage is surprising and counterintuitive, given that the MCP gene is highly expressed and essential for virion assembly. Since phage 1/4 infection proceeds rapidly and yields high numbers of progeny virions, it dictates that the virus can somehow bridge this translational discrepancy.

Consequently, we hypothesized that phage 1/4 infection might lead to changes in host tRNA modification levels that benefit viral replication, possibly by modulating the interaction between preferred codon–anticodon pairs and by expanding the coding capacity of the available tRNAs. To elucidate this, we carried out a comprehensive analysis of the tRNA modificome throughout the infection cycle. This revealed a complex pattern of tRNA modification dynamics (Figure 3A), which we grouped into four categories based on PTMs that showed statistically significant changes (Figure 3B–E). First, we note a subtle temporal correlation between the upregulation of ct^6^A at the early infection stage (Figure 3C) and the preference for A-ending serine and arginine codons in viral early-infection-stage genes (Table 1). Notably, t^6^A and its derivates, such as ct^6^A, promote accurate decoding of ANN codons [44], in addition to which t^6^A prevents leaky scanning of initiation codons and read-through of stop codons [45]—a strategy exploited by many viruses to regulate their gene expression at different infection stages [46,47]. Second, we also observed a temporal correlation between the increase in Q modification levels at the late infection stage (Figure 3D) and the strong codon bias towards tyrosine (TAC) in the viral late-infection-stage genes (Table 1). Intriguingly, the Q synthesis pathway is highly specific, and substrates are limited to G_34_-bases of GUN-anticodon tRNAs [6]. For tyrosine-carrying tRNAs, this requirement only matches tRNA$\begin{array}{c} \mathrm{GUA}\\ \mathrm{Tyr} \end{array}$, which decodes tyrosine (TAC) and aligns with the codon preference for phage 1/4 late genes. This suggests that Q modification may enhance the translation of late-infection-stage viral transcripts. Furthermore, our observation is supported by a recent study on mitochondrial translation, where changes in Q modification levels are tied to the regulation of codon-biased translation [48]. Therefore, this suggests that modulation of tRNA modifications in general, and of Q in particular, might constitute a translation control strategy employed by phage 1/4.

To the best of our knowledge, this constitutes the first study where tRNA modification changes are quantitatively characterized throughout a viral infection cycle. We show that tRNA modifications are dynamic and continuously respond to the various stages of phage 1/4 infection, although the exact mechanisms by which PTM levels are influenced remain to be determined. PTM biosynthesis often involves complex metabolic pathways that rely on the interplay of cellular metabolites and co-enzymes [3]. Phage 1/4 does not encode for RNA modification enzymes of its own [16], but it is nonetheless plausible that cellular metabolites and enzymes may be potential targets during phage 1/4 infection, as modulating their availability and activity would have a direct impact on tRNA modification levels. Hence, it is conceivable that tRNA modifications play interconnected roles in regulating the translation of host- and virus-derived proteins, as well as in other non-canonical regulatory functions [32]. Moreover, we uncovered a surprising codon usage bias for a set of late viral genes (Table 1), which ought to hamper their translation. However, phage 1/4 abrogates this challenge, potentially via a compensatory Q modification level change that may further codon-biased translation in a similar fashion to what was previously observed for mitochondria [48]. Taken together, this work provides the basis for further exploration of tRNA modification-mediated translational control during infection.

## Figures and Tables

**Table 1:** Preferred codons that were utilized differently between the host genome, virus genome, the set of early- or late-infection-stage genes, and the viral major capsid protein (MCP) gene.

Amino Acid	Host Genome	Virus Genome	Early Gene Set	Late Gene Set	Viral MCP Gene	Host Encodes	Virus Encodes
Alanine	GCA	GCT	GCA	GCT	GCT	3 × GCA 2 × GCC	
Cysteine	TGC	TGT	TGT	TGT	TGT	1 × TGC	
Phenylalanine	TTT	TTT	TTT	TTT	TTC	2 × TCC	
Isoleucine	ATT	ATT	ATT	ATA	ATC	4 × ATC	
Leucine	TTA	TTA	TTA	TTA/ CTA	CTA	1 × TGG 3 × TTA 2 × CTA 1 × CTC	
Lysine	AAA	AAA	AAA	AAG	AAA	9 × AAA	
Proline	CCA	CCT	CCA/ CCT	CCT	CCA	3 × CCA 1 × CCC	
Arginine	AGA	AGA	AGA	CGT	CGT	1 × AGA 1 × CGG 4 × CGC	1 × AGA
Serine	AGC/TCA	AGT	AGT	TCT	TCA	2 × AGC 2 × TCA 1 × TCC	
Threonine	ACA/ ACT/ACC	ACA	ACA/ ACT	ACT	ACT	2 × ACA 1 × ACC	
Valine	GTT	GTA/ GTT	GTT	GTT	GTT	6 × GTA 2 × GTC	
Tyrosine	TAT	TAT	TAT	TAC	TAC	4 × TAC	

## Data Availability

The data presented in this study are available in the Supplementary Materials Section (Lampi_etal_mass-spec-dataset.xlsx).

## Supplementary Materials

The following supporting information can be downloaded at: https://www.mdpi.com/article/10.3390/microorganisms11020355/s1, Supplementary Figure S1 Uniform Shewanella phage 1/4 infection of *Shewanella glacialimarina* is achieved with high virus concentrations, Supplementary Figure S2 Shewanella phage 1/4 infection trials with *S. baltica* and *S. frigidimarina*, Supplementary Figure S3 Change in extracellular and intracellular protein patterns of *S. glacialimarina* liquid cultures during phage 1/4 infection, Supplementary Figure S4 Phage 1/4 infection-induced intracellular changes in *Shewanella glacialimarina* cells, Supplementary Table S1 Codon usage of the *S. glacialimarina* and phage 1/4 genomes and selected viral genes.
