# Supplementary material for: Bacteriophage Infection of the Marine Bacterium Shewanella glacialimarina Induces Dynamic Changes in tRNA Modifications
Source: Microorganisms. 2023 Jan 31;11(2):355. doi: 10.3390/microorganisms11020355 (PMC9963407; doi:10.3390/microorganisms11020355)
Supplement: Supplementary file 1 [file microorganisms-11-00355-s001.zip › microorganisms-2146690-supplementary.pdf]

microorganisms

Supplementary materials

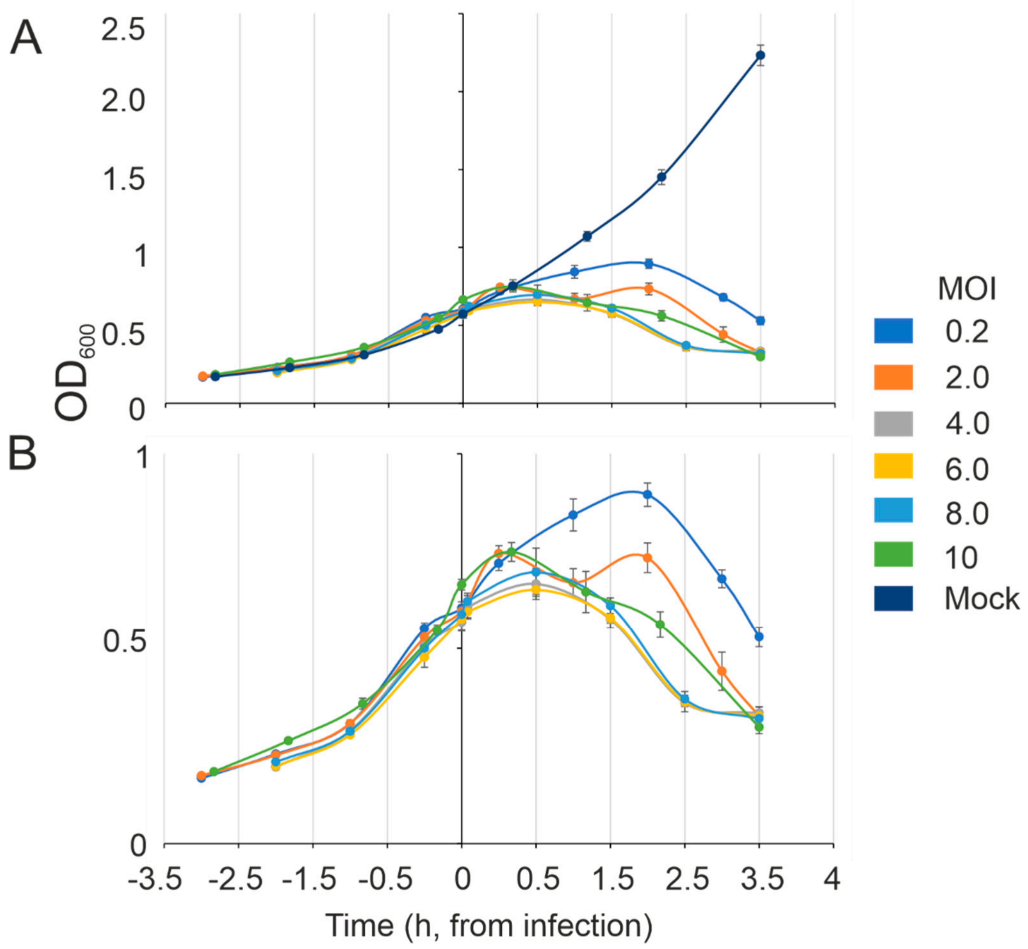

Figure S1. Uniform Shewanella phage 1/4 infection of *Shewanella glacialimarina* is achieved with high virus concentrations. (A) Growth curves for *S. glacialimarina* infected with phage 1/4 at multiplicity of infection (MOI) values of 0.2-10 compared to mock-infected *S. glacialimarina*. (B) Close-up of phage 1/4 infected *S. glacialimarina* growth curves. Error bars indicate the standard deviation (n=3).

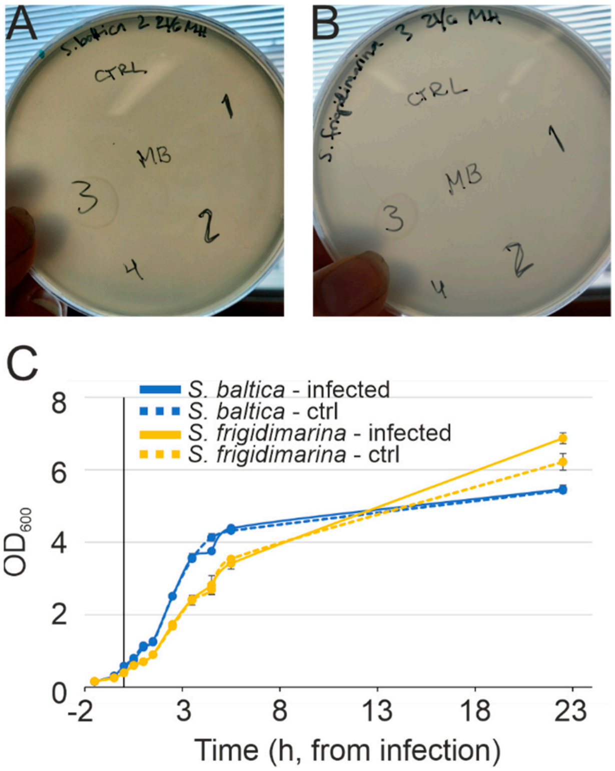

Figure S2. Shewanella phage 1/4 infection trials with *S. baltica* and *S. frigidimarina.* Spot-on-lawn assay using (A) *S. baltica*, and (B) *S. frigidimarina.* Bacterial lawns were infected with virus stocks of different purity: 1=agar stock, 2=PEG-supernatant, 3= PEG-pellet, 4= 1 × virus, and CTRL= 25% rMB. (C) Growth curves of both potential hosts in 25% rMB upon addition of Shewanella phage 1/4 at MOI 10. Ctrl= mock infected culture. Error bars depict the standard deviation (n=3).

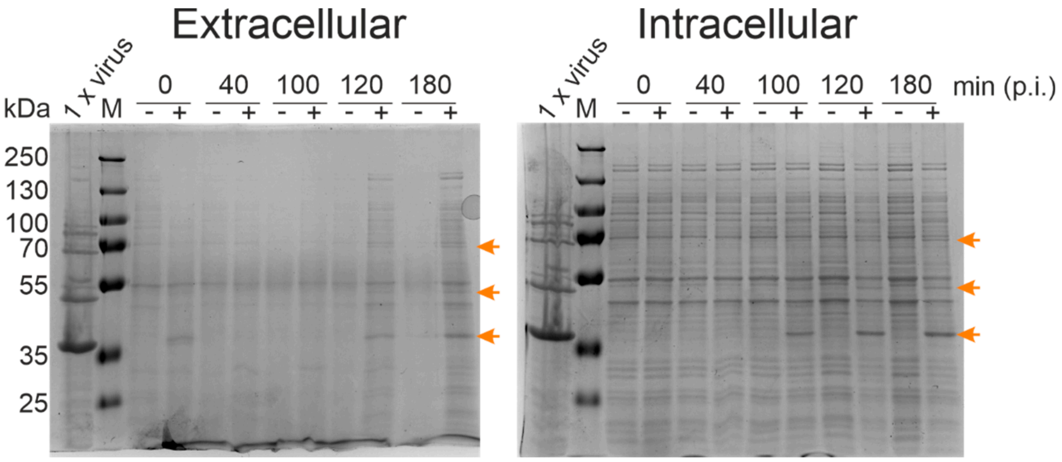

Figure S3. Change in extracellular and intracellular protein patterns of *S. glacialimarina* liquid cultures during phage 1/4 infection. Protein samples were collected at 0, 40, 100, 120, and 180 min post infection (p.i.) from either MOI 10 infected (+) or mock-infected (-) cultures. Extracellular viral proteins appear in the growth media at 120 min p.i. (indicated with arrows), whereas intracellular viral proteins are observed already at 100 min p.i. The phage 1/4 1 × virus stock is included as a control to aid in the identification of viral proteins. M= size marker, kDa indicated on the left.

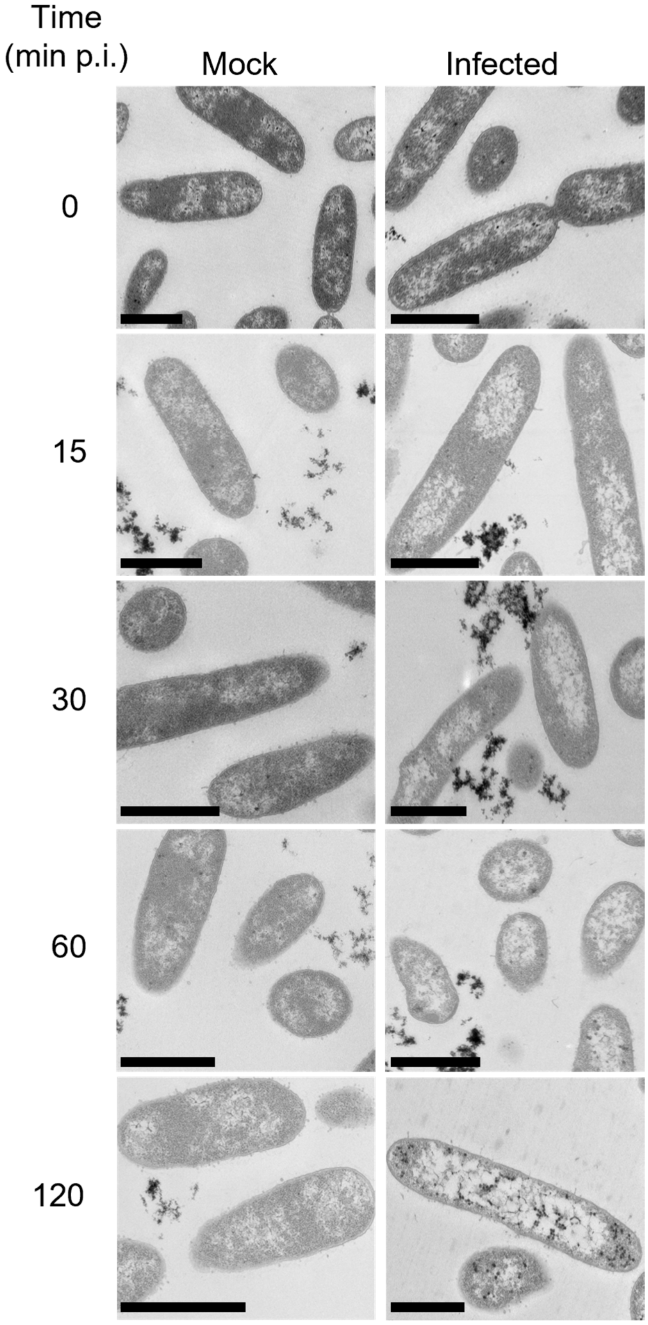

Figure S4. Phage 1/4 infection-induced intracellular changes in *Shewanella glacialimarina* cells. TEM micrographs of thin-cut preparates of infected and mock-infected cells at 0, 15, 30, 60, and 120 min p.i. Size bar= 1 μm.

Table S1. Codon usage of the *S. glacialimarina* and phage 1/4 genomes and selected viral genes.

**Amino acid**	**codon**	**HOST**	**VIRUS**	**VIRUS, early gene set**	**VIRUS, late gene set**	**VIRUS, MCP**	**HOST encodes**	**VIRUS encodes**
		Fraction (Number)						
**Ala**	GCG	0.2 (17006)	0.07 (147)	0.08 (6)	0.07 (7)	0.06 (2)		

	GCA	**0.31 (26535)**	0.39 (793)	**0.51 (39)**	0.41 (43)	0.44 (16)	3	
	GCT	0.28 (24569)	**0.43 (863)**	0.3 (23)	**0.47 (49)**	**0.5 (18)**		
	GCC	0.22 (18770)	0.10 (208)	0.11 (8)	0.06 (6)	0 (0)	2	
**Cys**	TGT	0.47 (18394)	**0.78 (456)**	**0.88 (21)**	**0.67 (4)**	**1 (1)**		
	TGC	**0.53 (21056)**	0.22 (126)	0.13 (3)	0.33 (2)	0 (0)	1	
**Asp**	GAT	**0.68 (30987)**	**0.73 (1763)**	**0.67 (68)**	**0.63 (43)**	**0.53 (10)**		
	GAC	0.32 (14458)	0.27 (668)	0.33 (33)	0.37 (25)	0.47 (9)	3	
**Glu**	GAG	0.32 (12945)	0.37 (865)	0.36 (38)	0.35 (28)	0.33 (8)		
	GAA	**0.68 (27837)**	**0.63 (1496)**	**0.64 (67)**	**0.65 (52)**	**0.67 (16)**	6	
**Phe**	TTT	**0.74 (38773)**	**0.62 (903)**	**0.65 (33)**	**0.56 (32)**	0.43 (6)		
	TTC	0.26 (13326)	0.38 (548)	0.35 (18)	0.44 (25)	**0.57 (8)**	2	
**Gly**	GGG	0.15 (10121)	0.15 (379)	0.16 (17)	0.03 (2)	0 (0)		
	GGA	0.15 (9536)	0.18 (437)	0.11 (12)	0.21 (13)	0.04 (1)	1	1*
	GGT	**0.38 (25059)**	**0.58 (1442)**	**0.63 (67)**	**0.69 (42)**	**0.91 (21)**		
	GGC	0.32 (20950)	0.09 (217)	0.09 (10)	0.07 (4)	0.04 (1)	5	
**His**	CAT	**0.61 (20074)**	**0.56 (424)**	**0.76 (29)**	**0.65 (31)**	**0.5 (4)**		
	CAC	0.39 (12926)	0.44 (328)	0.24 (9)	0.35 (17)	**0.5 (4)**	2	
**Ile**	ATA	0.23 (18389)	0.35 (845)	0.37 (29)	**0.38 (28)**	0 (0)		
	ATT	**0.53 (41868)**	**0.46 (1117)**	**0.44 (35)**	0.31 (23)	0.4 (6)		
	ATC	0.23 (18139)	0.18 (442)	0.19 (15)	0.31 (23)	**0.6 (9)**	4	
**Lys**	AAG	0.36 (24203)	0.38 (1013)	0.42 (56)	**0.55 (84)**	0.43 (3)		
	AAA	**0.64 (43438)**	**0.62 (1633)**	**0.58 (76)**	0.45 (70)	**0.57 (4)**	9	
**Leu**	TTG	0.20 (25041)	0.15 (469)	0.12 (16)	0.14 (28)	0.11	1	

						(3)		
	TTA	**0.33 (41154)**	**0.42 (1344)**	**0.43 (59)**	**0.28 (55)**	0.19 (5)	3	
	CTG	0.13 (16176)	0.06 (190)	0.02 (3)	0.16 (31)	0.04 (1)		
	CTA	0.12 (14832)	0.18 (583)	0.14 (20)	**0.30 (59)**	**0.41 (11)**	2	
	CTT	0.16 (20672)	0.15 (486)	0.25 (34)	0.11 (21)	0.19 (5)		
	CTC	0.07 (8477)	0.04 (129)	0.04 (6)	0.02 (4)	0.07 (2)	1	
**Met**	ATG	**1 (27779)**	**1 (1038)**	**1 (35)**	**1 (54)**	**1 (9)**	7	
**Asn**	AAT	**0.59 (33309)**	**0.61 (1287)**	**0.64 (38)**	0.37 (28)	0.06 (1)		
	AAC	0.41 (23081)	0.39 (814)	0.36 (21)	**0.63 (47)**	**0.94 (16)**	5	
**Pro**	CCG	0.21 (10515)	0.05 (59)	0.02 (1)	0.03 (1)	0 (0)		
	CCA	**0.36 (18044)**	0.38 (429)	**0.46 (26)**	0.33 (13)	**0.56 (5)**	3	
	CCT	0.26 (13354)	**0.48 (543)**	**0.48 (27)**	**0.59 (23)**	0.44 (4)		
	CCC	0.17 (8528)	0.08 (91)	0.04 (2)	0.05 (2)	0 (0)	1	
**Gln**	CAG	0.38 (20308)	0.33 (394)	0.27 (11)	**0.53 (52)**	0.31 (5)		
	CAA	**0.62 (33428)**	**0.67 (802)**	**0.73 (30)**	0.47 (47)	**0.69 (11)**	4	
**Arg**	AGC	0.15 (12682)	0.21 (301)	0.26 (16)	0.18 (17)	0 (0)		
	AGA	**0.22 (18674)**	**0.33 (483)**	**0.40 (25)**	0.30 (28)	0 (0)	1	1**
	CGG	0.11 (9561)	0.03 (39)	0.02 (1)	0.03 (3)	0 (0)	1	
	CGA	0.16 (13034)	0.08 (120)	0.03 (2)	0.06 (6)	0 (0)		
	CGT	0.18 (14910)	0.30 (433)	0.24 (15)	**0.37 (34)**	**0.88 (22)**	4	
	CGC	0.18 (14981)	0.05 (79)	0.05 (3)	0.05 (5)	0.12 (3)		
**Ser**	AGT	0.21 (21750)	**0.33 (836)**	**0.39 (34)**	0.25 (30)	0.04 (1)		
	AGC	**0.24 (24190)**	0.08 (196)	0.10 (9)	0.07 (9)	0.13 (3)	2	

	TCG	0.12 (12071)	0.04 (92)	0 (0)	0.03 (3)	0 (0)	
	TCA	**0.24 (24022)**	0.22 (558)	0.18 (16)	0.23 (27)	**0.43 (10)**	2
	TCT	0.12 (12046)	0.28 (729)	0.26 (23)	**0.31 (38)**	0.39 (9)	
	TCC	0.07 (7322)	0.06 (153)	0.06 (5)	0.12 (14)	0 (0)	1
**Thr**	ACG	0.16 (11199)	0.06 (161)	0.04 (3)	0.15 (17)	0 (0)	
	ACA	**0.29 (20103)**	**0.41 (1043)**	**0.42 (32)**	0.30 (35)	0.12 (3)	2
	ACT	**0.28 (19239)**	0.38 (955)	**0.44 (34)**	**0.36 (42)**	**0.8 (20)**	
	ACC	**0.27 (18242)**	0.14 (361)	0.10 (8)	0.20 (23)	0.08 (2)	1
**Val**	GTG	0.26 (19867)	0.18 (468)	0.19 (20)	0.23 (31)	0.12 (3)	
	GTA	0.22 (16478)	**0.38 (1020)**	0.32 (34)	0.32 (43)	0.32 (8)	6
	GTT	**0.36 (26898)**	**0.37 (976)**	**0.39 (41)**	**0.37 (49)**	**0.52 (13)**	
	GTC	0.16 (12313)	0.07 (193)	0.1 (10)	0.08 (11)	0.04 (1)	2
**Trp**	TGG	**1 (23833)**	**1 (584)**	**1 (23)**	**1 (12)**	**1 (3)**	1
**Tyr**	TAT	**0.65 (31241)**	**0.62 (960)**	**0.67 (38)**	0.47 (21)	0.38 (5)	
	TAC	0.35 (16574)	0.38 (593)	0.33 (19)	**0.53 (24)**	**0.62 (8)**	4
**STOP**	TGA	**0.43 (28980)**	0.14 (30)	**0.67 (2)**	0.11 (5)	0 (0)	
	TAG	0.19 (13258)	0.23 (50)	0 (0)	**0.54 (25)**	0 (0)	
	TAA	0.38 (25840)	**0.64 (142)**	0.33 (1)	0.35 (16)	**1 (1)**	

*Virus-encoded isoacceptor for glycine

**Virus-encoded isodecoder for arginine; the body of the tRNA is similar to lysine, the anticodon matches arginine. Preferred codons bolded.
